# Silica Nanoparticles Promote Apoptosis in Ovarian Granulosa Cells via Autophagy Dysfunction

**DOI:** 10.3390/ijms24065189

**Published:** 2023-03-08

**Authors:** Zhen Zheng, Wenlong Zuo, Rongrong Ye, Jason William Grunberger, Nitish Khurana, Xianyu Xu, Hamidreza Ghandehari, Fenglei Chen

**Affiliations:** 1 College of Veterinary Medicine, Yangzhou University, Yangzhou 225009, China; 2 Jiangsu Co-Innovation Center for Prevention and Control of Important Animal Infectious Diseases and Zoonoses, Yangzhou 225009, China; 3 Joint International Research Laboratory of Agriculture and Agri-Product Safety of the Ministry of Education of China, Yangzhou University, Yangzhou 225009, China; 4 Department of Molecular Pharmaceutics, University of Utah, Salt Lake City, UT 84112, USA; 5 Utah Center for Nanomedicine, University of Utah, Salt Lake City, UT 84112, USA; 6 Department of Biomedical Engineering, University of Utah, Salt Lake City, UT 84112, USA

**Keywords:** autophagy dysfunction, apoptosis, pvarian granulosa cells, lysosomal impairment, silica nanoparticles

## Abstract

Although silica nanoparticles (SNPs) are generally thought to be biocompatible and safe, the adverse effects of SNPs were also reported in previous studies. SNPs cause follicular atresia via the induction of ovarian granulosa cell apoptosis. However, the mechanisms for this phenomenon are not well understood. This study focuses on exploring the relationship between autophagy and apoptosis induced by SNPs in ovarian granulosa cells. Our results showed that 25.0 mg/kg body weight (b.w.)/intratracheal instillation of 110 nm in diameter spherical Stöber SNPs caused ovarian granulosa cell apoptosis in follicles in vivo. We also found that SNPs mainly internalized into the lumens of the lysosomes in primary cultured ovarian granulosa cells in vitro. SNPs induced cytotoxicity via a decrease in viability and an increase in apoptosis in a dose-dependent manner. SNPs increased BECLIN-1 and LC3-II levels, leading to the activation of autophagy and increased P62 level, resulting in the blockage of autophagic flux. SNPs increased the BAX/BCL-2 ratio and cleaved the caspase-3 level, resulting in the activation of the mitochondrial-mediated caspase-dependent apoptotic signaling pathway. SNPs enlarged the LysoTracker Red-positive compartments, decreased the CTSD level, and increased the acidity of lysosomes, leading to lysosomal impairment. Our results reveal that SNPs cause autophagy dysfunction via lysosomal impairment, resulting in follicular atresia via the enhancement of apoptosis in ovarian granulosa cells.

## 1. Introduction

With advantages of diverse synthetic approaches resulting in highly tunable physicochemical properties, high stability, large specific surface area, strong adsorption capacity, and good biocompatibility, synthetic silica nanoparticles (SNPs) have been widely applied for industrial and household applications and clinical purposes, such as food, cosmetics, and pharmaceutical products [1]. Due to the large-scale production and wide application, these anthropogenic SNPs enter the atmospheric environment as fine particles (PM_2.5_), resulting in the concentration increase in the environmental pollution [2]. As a result of their small size and large surface area, SNPs exhibit unique bioactivities and interactions with cellular or subcellular structures [3,4]. A previous study has found that SNPs (90 nm, amorphous, 21.0 mg/kg body weight (b.w.) enter the blood circulation and accumulate in the ovary, leading to follicular atresia by intratracheal instillation [5]. SNP-induced follicular atresia is mainly caused by apoptosis in ovarian granulosa cells [5]. Ovarian granulosa cells are one of the main components in the follicles and cooperate in their development. However, the mechanism of apoptosis induced by SNPs in ovarian granulosa cells is not well known.

Previous investigations have revealed several possible toxicological mechanisms for SNP-induced cytotoxicity, such as oxidative stress and autophagy. SNP (110 nm, spherical, 100 μg/mL) exposure causes the excessive generation of ROS, leading to oxidative stress-induced toxicity in human hepatocellular carcinoma HepG2 cells [6] and human lung adenocarcinoma cells (A549) [7]. SNPs (107 nm, spherical, 20 mg/kg) decrease sperm quantity and quality, and damage both mitochondria and DNA in spermatogenic cells in male reproductive toxicology [8]. In addition, SNPs (90 nm, amorphous, 21 mg/kg b.w.) induce ovarian granulosa cell apoptosis, resulting in follicular atresia in female reproductive toxicology [5] via oxidative stress. Autophagy is a protective mechanism that recycles damaged organelles and degrades long-lived proteins to maintain cellular homeostasis [9]. Oxidative stress induces autophagy, resulting in apoptosis [10]. Our recent studies show that SNPs (110 nm, spherical) reduce the population of Leydig cells and disorganization of spermatogenic cell layers under 25 mg/kg dose in vivo, enhance testosterone secretion under 100 μg/mL [11], and suppress Leydig cell apoptosis via the activation of caspase-8 under 400 μg/mL in vitro [12]. Autophagy dysfunction results in the accumulation of impaired organelles and unfolded/misfolded proteins, ultimately leading to cell death [13,14]. SNPs have been reported to cause autophagy dysfunction via the blockage of autophagic flux and the induction of lysosomal impairment in HeLa cells [15], human lung bronchial epithelial (BEAS-2B) cells [14], and hepatocytes [13]. In addition to oxidative stress and autophagy, Duan et al. found that SNPs trigger hepatic lipid metabolism disorder via the TLR5-signaling pathway [16]. Increasing numbers of molecular mechanisms were reported concerning the toxicity of SNPs. However, the effects of autophagy induced by SNPs on granulosa cell apoptosis need to be further explored.

Since the toxicity of SNPs is always a function of dose, the physicochemical properties, and the route of administration, we mainly focus on the impacts of the dose-dependence of toxicity in ovarian granulosa cells. To better explore the mechanisms of follicular atresia induced by SNPs, our study aimed to investigate the relationship between autophagy and apoptosis induced by SNPs in ovarian granulosa cells. This study provides new evidence for female reproductive toxicity induced by SNPs.

## 2. Results

### 2.1. Characterization of SNPs

The images of transmission electron microscopy (TEM) showed that the particles of SNPs exhibited low polydispersity and were nearly spherical in shape (Figure S1A) with an average size of 111.6 ± 14.0 nm in diameter (Figure S1B). The representative hydrodynamic diameter and zeta potential in 10 mM NaCl solution were 110.8 ± 26.5 nm and −49.7 ± 26.5, as reported by our previous study [11].

### 2.2. Effect of SNPs on Ovarian Structure In Vivo

H and E and TUNEL staining were performed to assess apoptosis in the ovary. H and E staining results showed that normal follicle development and compact and regular arrangement of granulosa cells were observed in the ovaries of the control and the 12.5 mg/kg SNP groups (Figure S2A,B). However, in the 25.0- and 50.0-mg/kg SNP groups, unordered and scattered granulosa cells in the granulosa layers were increased (Figure S2C,D). There were more secondary follicles, early antral follicles, corpus luteum, and atretic follicles in the ovaries of the 25.0- and 50.0-mg/kg SNP groups (Figure S2C,D). TUNEL staining results showed that, compared to the control group (Figure S2E,F), the number of TUNEL-positive cells, which glowed a bright green fluorescence, significantly increased in the SNP group (Figure S2G,H).

### 2.3. SNPs were Internalized into the Lysosomes in Ovarian Granulosa Cells

**Figure 1:**
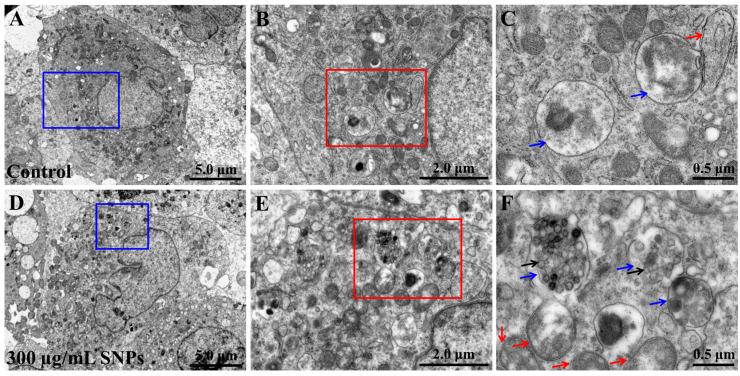
Cellular uptake of SNPs. (**A**) TEM image in the control group. (**B**) Blue area in (**A**). (**C**) Red area in (**B**). (**D**) TEM image in the SNP group. Primary cultured ovarian granulosa cells were exposed to 300 μg/mL SNPs for 24 h. (**E**) Blue area in (**D**). (**F**) Red area in (**E**). Black arrows indicate SNPs. Blue arrows indicate monolayer membranous vesicles containing lysosomes. Red arrows indicate bilayer membranous vesicles containing autophagosomes.

The TEM results showed that no particles were detected in primary cultured ovarian granulosa cells of the control group (Figure 1A–C). However, in the SNP group, there were numerous particles in granulosa cells, which were mainly localized in monolayer membranous vesicles containing lysosomes (Figure 1D–F) although there were more bilayer vesicles containing autophagosomes (Figure 1F).

### 2.4. SNPs Induced Cytotoxicity in Ovarian Granulosa Cells

**Figure 2:**
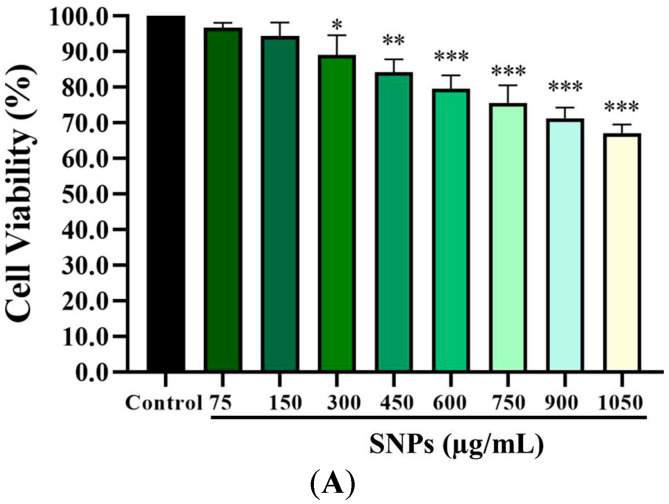

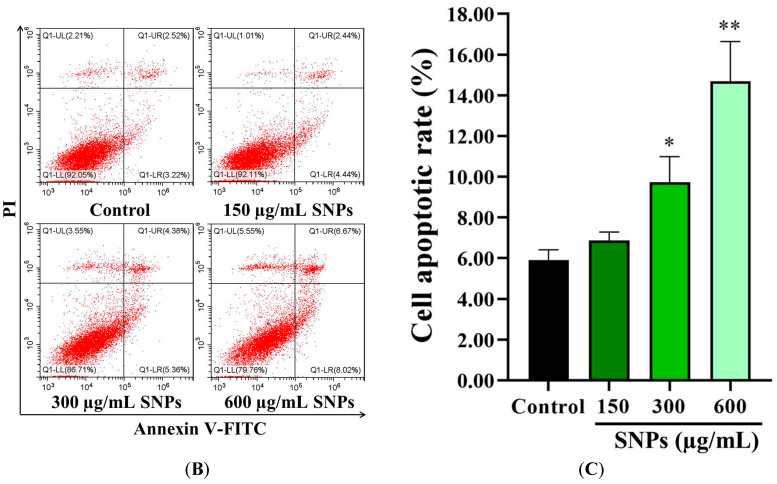
Cytotoxicity of SNPs in primary cultured granulosa cells. (**A**) Cell viability was measured by CCK-8 after 0, 75, 150, 300, 450, 600, 750, 900, and 1050 µg/mL SNP exposures for 24 h. (**B**) The apoptotic rate was determined by flow cytometry after 0, 150, 300, and 600 µg/mL SNP exposures for 24 h. (**C**) Quantification of the apoptotic rate in (**B**). * *p* < 0.05, ** *p* < 0.01, and *** *p* < 0.001 vs. the control.

The CCK-8 assay results showed that lower concentrations (0–150 µg/mL) of SNPs had no significant impact on cell viability (Figure 2A), while the cell viability was significantly decreased at higher concentrations (300–1050 µg/mL, Figure 2A), indicating a dose-dependent cytotoxicity. In addition, the flow cytometry results showed that the apoptotic rate was significantly increased at (5.74 ± 0.50)%, (6.88 ± 0.40)%, (9.74 ± 1.25)%, and (14.69 ± 1.95)% after 0, 150, 300, and 600 µg/mL SNP exposures, respectively (Figure 2B,C).

### 2.5. SNPs Caused Autophagy Dysfunction in Ovarian Granulosa Cells

**Figure 3:**
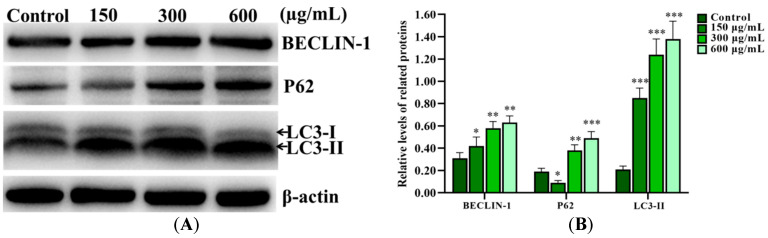

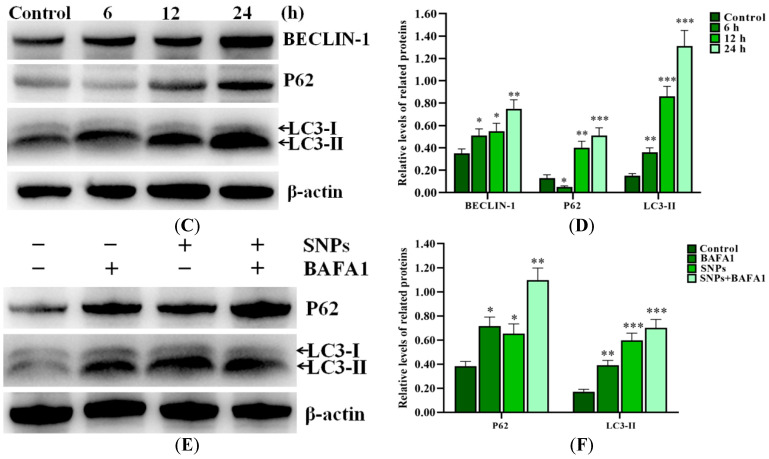
Activation of SNPs on autophagy in ovarian granulosa cells. (**A**) Autophagy-related proteins were analyzed after 0, 150, 300, and 600 μg/mL SNP exposures for 12 h. (**B**) Densitometric analysis of BECLIN-1, P62, and LC3-II levels in (**A**). (**C**) Autophagy-related proteins were analyzed after 300 μg/mL SNP exposure for 0, 6, 12, and 24 h. (**D**) Densitometric analysis of BECLIN-1, P62, and LC3-II levels in (**C**). (**E**) The levels of P62 and LC3-II were analyzed after 300 μg/mL SNP exposure for 12 h in the absence or presence of 100 nM BAFA1. (**F**) Densitometric analysis of (**E**). * *p* < 0.05, ** *p* < 0.01, and *** *p* < 0.001 vs. the control.

Western blot results showed that SNPs significantly increased BCL-2-interacting protein (BECLIN-1) and microtubule-associated protein light chain 3 II (LC3-II) levels in a dose-dependent manner and also increased the Sequestosome 1 (SQSTM1/P62) level after 300 and 600 µg/mL SNP exposures for 12 h, while they decreased the P62 level after 150 µg/mL SNP exposure compared to the control group (Figure 3A,B). In addition to an increase in the BECLIN-1 and LC3-II levels, SNPs also significantly increased the P62 level at 12 and 24 h, while it decreased at 6 h after 300 µg/mL SNP exposure (Figure 3C,D). Furthermore, SNPs, combined with Bafilomycin A1 (BAFA1), significantly increased the P62 and LC3-II levels compared to the SNP-only group (Figure 3E,F).

### 2.6. SNPs Induced Apoptosis via the Activation of the Mitochondrial Pathway

**Figure 4:**
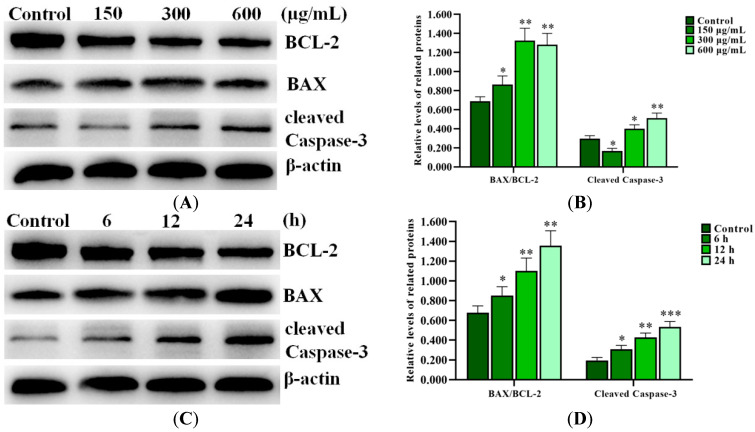
Activation of SNPs on the mitochondrial apoptotic pathway in ovarian granulosa cells. (**A**) The apoptotic proteins were analyzed after 0, 150, 300, and 600 μg/mL SNP exposures for 12 h. (**B**) Densitometric analysis of the BAX/BCL-2 ratio and cleaved caspase-3 level in (**A**). (**C**) The apoptotic proteins were analyzed after 300 μg/mL SNP exposure for 0, 6, 12, and 24 h. (**D**) Densitometric analysis of the BAX/BCL-2 ratio and cleaved caspase-3 level in (**C**). * *p* < 0.05, ** *p* < 0.01, and *** *p* < 0.001 vs. the control.

Western blot results showed that SNPs significantly increased the BAX/BCL-2 ratio and the cleaved caspase-3 level (Figure 4A,B). BCL-2, BAX, and cleaved caspase-3 levels were also investigated after 300 µg/mL SNP exposure at different times, respectively. Western blot results showed that SNPs significantly increased the BAX/BCL-2 ratio and the cleaved caspase-3 level in a time-dependent manner (Figure 4C,D).

### 2.7. Autophagy Dysfunction Enhanced SNP-Induced Apoptosis in Ovarian Granulosa Cells

**Figure 5:**
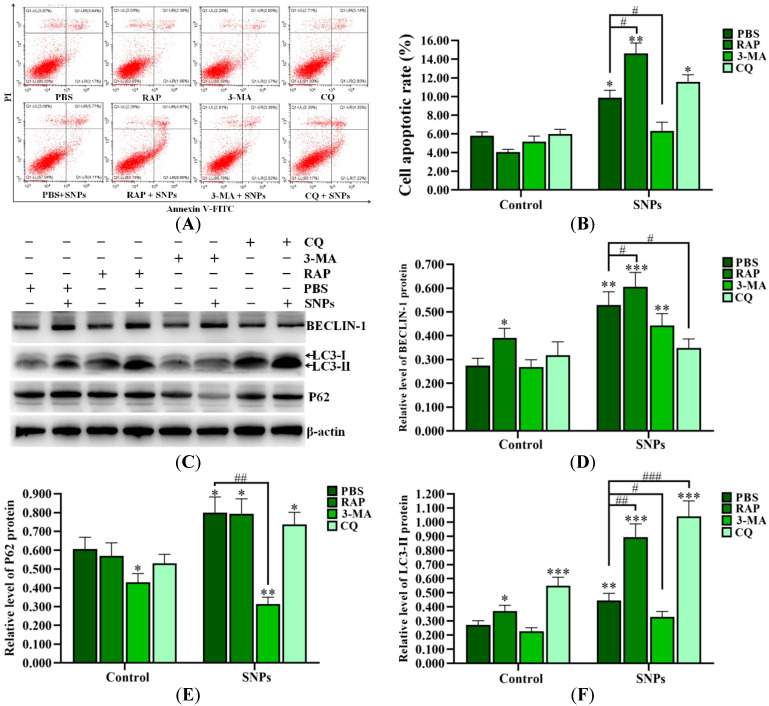

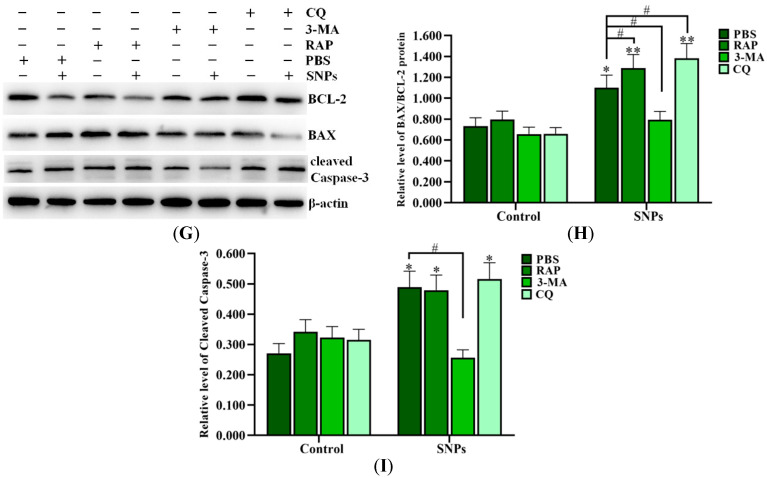
Inhibition of autophagy upon apoptosis induced by SNPs. (**A**) Cytotoxicity was determined by flow cytometry after 300 µg/mL SNP exposure, combined with 0.01 M PBS, 1 µM RAP, 0.2 µM 3-MA, and 5 µM CQ for 24 h. (**B**) Quantification of the apoptotic rate in (**A**). (**C**) Autophagy-related proteins were analyzed by Western blot. (**D**) Densitometric analysis of the BECLIN-1 level in (**C**). (**E**) Densitometric analysis of the P62 level in (**C**). (**F**) Densitometric analysis of the LC3-II level in (**C**). (**G**) The apoptotic proteins were analyzed by Western blot. (**H**) Densitometric analysis of the BAX/BCL-2 ratio in (**G**). (**I**) Densitometric analysis of cleaved caspase-3 level in (**G**). * *p* < 0.05, ** *p* < 0.01, and *** *p* < 0.001 vs. the control; # *p* < 0.05, ## *p* < 0.01, and ### *p* < 0.001 vs. SNP + PBS group.

SNPs, combined with PBS (control group), 3-methyladenine (3-MA), chloroquine (CQ), and rapamycin (Rap), were exposed to ovarian granulosa cells. The flow cytometry results showed that the apoptotic rates in the SNP + RAP and SNP + CQ groups (14.62 ± 1.12)% and (11.57 ± 0.76)%, respectively, were increased, while they were significantly decreased in the SNP + 3-MA group (6.30 ± 0.95)%) compared to the SNP + PBS group (9.88 ± 0.80)%) after SNP exposure (Figure 5A,B). Meanwhile, Western blot results showed that SNPs, combined with RAP, significantly increased BECLIN-1 and LC3-II levels and had no significant difference in the P62 level compared to the SNP + PBS group. Combination with CQ significantly decreased the BECLIN-1 level, increased the LC3-II level, and had no significant difference on the P62 level compared to the SNP + PBS group. Combination with 3-MA had no significant difference on the BECLIN-1 level and decreased the LC3-II and P62 levels compared to the SNP + PBS group (Figure 5C–F). Furthermore, SNPs, combined with RAP or CQ, significantly increased the BAX/BCL-2 ratio, and had no significant difference on the cleaved caspase-3 level (Figure 5G,H), while combination with 3-MA significantly decreased the BAX/BCL-2 ratio and the cleaved caspase-3 levels compared to the SNP + PBS group (Figure 5G,I).

### 2.8. BECLIN-1 Depletion Inhibited Apoptosis Induced by SNPs in Ovarian Granulosa Cells

**Figure 6:**
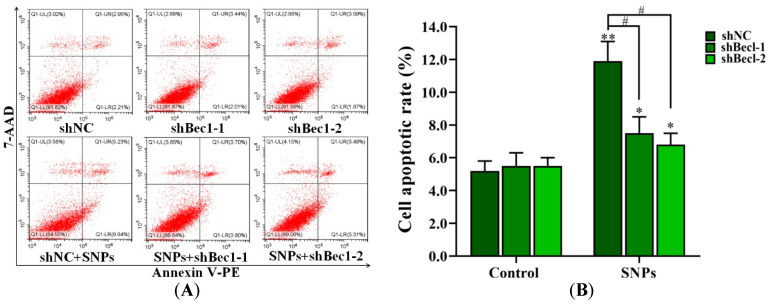

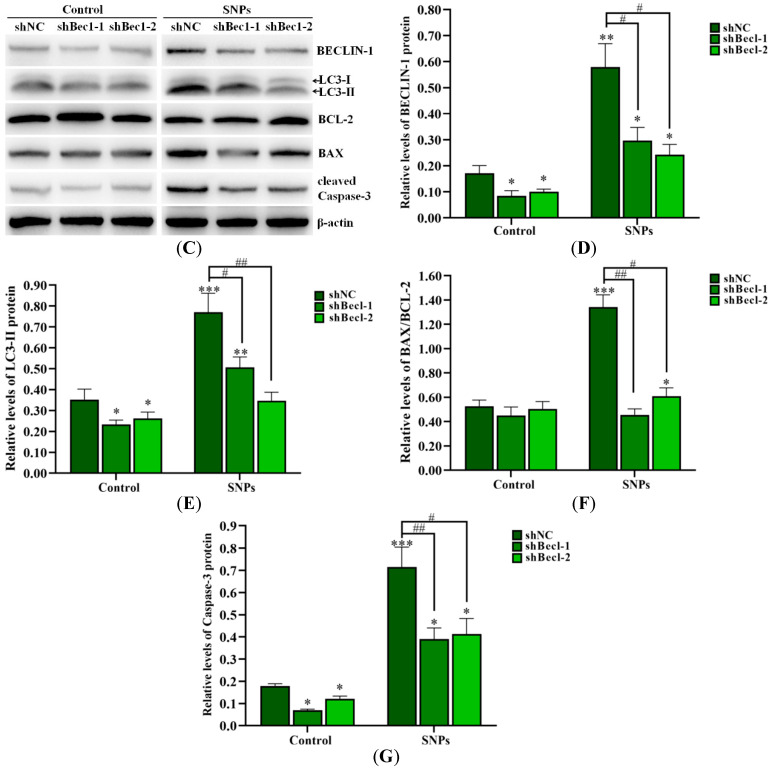
Knockdown of BECLIN-1 on apoptosis induced by SNPs in ovarian granulosa cells. (**A**) Cytotoxicity was determined by flow cytometry after 300 µg/mL SNP exposure in BECLIN-1 knockdown (shBecl-1 and shBecl-1) ovarian granulosa cells. (**B**) Quantification of the apoptotic rate in (**A**). (**C**) Autophagy-related and apoptotic proteins were analyzed by Western blot. (**D**) Densitometric analysis of BECLIN-1 level in (**C**). (**E**) Densitometric analysis of LC3-II level in (**C**). (**F**) Densitometric analysis of the BAX/BCL-2 ratio in (**C**). (**G**) Densitometric analysis of the cleaved caspase-3 level in (**C**). * *p* < 0.05, ** *p* < 0.01, and *** *p* < 0.001 vs. shNC group; # *p* < 0.05 and ## *p* < 0.01 vs. shNC + SNP group.

The flow cytometry results showed that, compared to the shNC + SNP group (11.87 ± 1.20)%), the apoptotic rates in the shBec1-1 + SNP and shBec1-2 + SNP groups (7.50 ± 0.96)% and (6.79 ± 0.68)%, respectively, were significantly decreased (Figure 6A,B). In addition, Western blot results showed that BECLIN-1, LC3-II, cleaved caspase-3 levels, and the BAX/BCL-2 ratio were significantly decreased in the shBec1-1 + SNP and shBec1-2 + SNP groups (Figure 6C–G).

### 2.9. Lysosome Impairment Induced by SNPs Blocked Autophagic Flux

**Figure 7:**
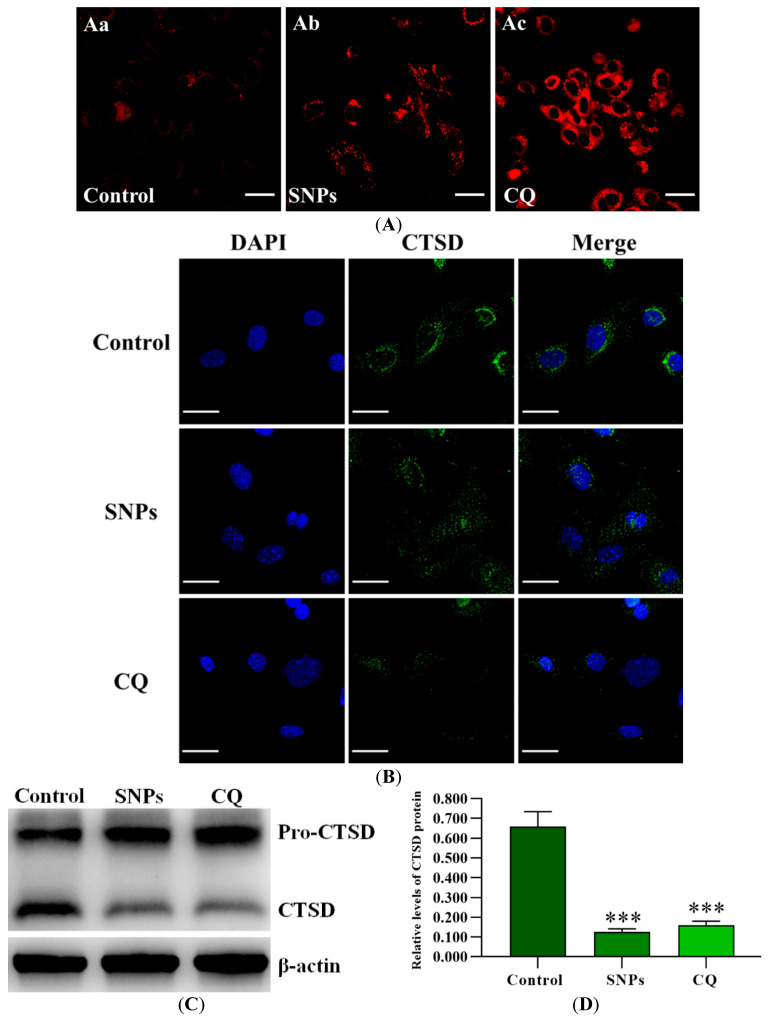
Lysosomal function after SNP exposure. (**A**) The acidity of lysosomes was qualitatively determined by fluorescence microscopy using LysoTracker Red dye after exposure to the control (Aa), 300 µg/mL SNPs (Ab), or 100 mM CQ (Ac) for 24 h. (**B**) The subcellular localization of CTSD was examined by fluorescence microscopy. (**C**) The level of CTSD in the lysosomes was analyzed by Western blot. (**D**) Densitometric analysis of mature CTSD level in C. *** *p* < 0.001 vs. the control.

To get more insight into the effect of SNPs on the alteration of lysosomal pH, the acidity of the lysosomes was qualitatively assessed using LysoTracker Red. Fluorescence microscopy results showed that SNPs significantly increased the area of LysoTracker Red-positive structures, which was consistent with the positive CQ group (Figure 7A). To examine the effect of SNPs on lysosomal integrity, the subcellular localization of Cathepsin D (CTSD) was assessed. Fluorescence microscopy results showed that cells in the control group displayed discrete and clumped green fluorescence, while the cells exposed to SNPs showed diffuse and cytoplasmic green fluorescence, which was consistent with the positive CQ group (Figure 7B). Furthermore, Western blot results showed that SNP significantly decreased the level of the mature form of CTSD in the lysosomes, which was also consistent with the CQ group (Figure 7C,D).

### 2.10. Inhibition of ROS Decreased SNP-Induced Autophagy and Apoptosis

**Figure 8:**
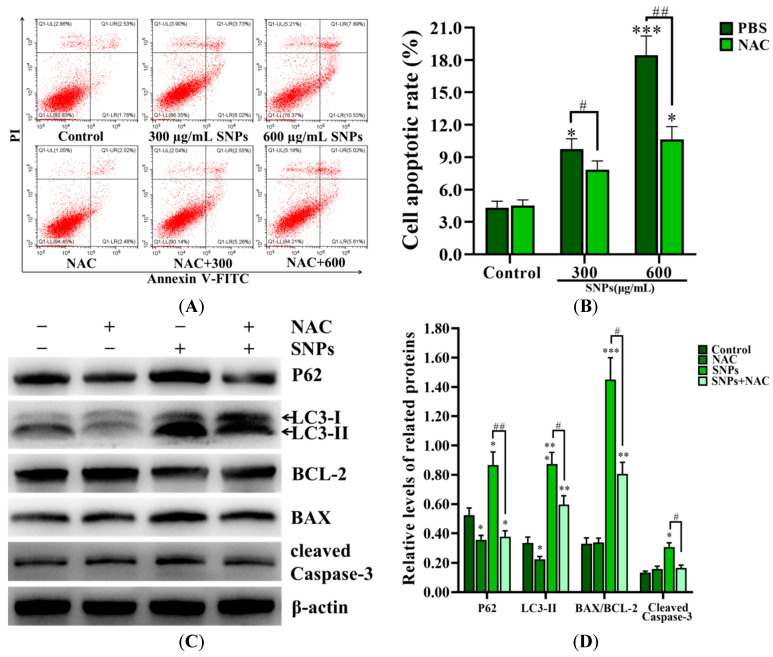
Effect of ROS on autophagy and apoptosis induced by SNPs. (**A**) Cytotoxicity was determined by flow cytometry after 300 and 600 µg/mL SNP exposures combined with 10 mM NAC for 24 h. (**B**) Quantification of the apoptotic rate in (**A**). (**C**) Autophagy-related and apoptotic proteins were analyzed by Western blot after 600 µg/mL SNP exposures, combined with 10 mM NAC for 12 h. (**D**) Densitometric analysis of the BAX/BCL-2 ratio, P62, LC3-II, and cleaved caspase-3 levels in (**C**). * *p* < 0.05, ** *p* < 0.01, and *** *p* < 0.001 vs. the control; # *p* < 0.05 and ## *p* < 0.01 vs. SNP + PBS group.

SNPs, combined with an antioxidant *N*-acetylcysteine (NAC), were exposed to granulosa cells to investigate the effect of ROS on the relationship between autophagy and apoptosis induced by SNPs. Flow cytometry results demonstrated that the apoptotic rate in the 300 µg/mL SNP + NAC group (7.83 ± 0.81)%) had no significant difference (9.75 ± 0.95)%) compared to the 300 µg/mL SNP group, while it was decreased in the 600 µg/mL SNP + NAC group (10.63 ± 1.20)%) compared to the 600 µg/mL SNP group (18.42 ± 1.80)%) (Figure 8A,B). Meanwhile, Western blot results showed that P62, LC3-II, cleaved caspase-3 levels, and the BAX/BCL-2 ratio were significantly decreased in the SNP + NAC group (Figure 8C,D).

## 3. Discussion

The large-scale production and wide application of SNPs have increased public concerns on the exposure risks to the environment [1]. Hundreds of metric tons of SNPs were released into water, soil, and landfill in Asia per year [17]. Despite generally being considered to be biocompatible, the adverse effects of SNPs were also reported in previous studies. In female reproductive toxicology, SNPs cause ovarian granulosa cell apoptosis, resulting in follicular atresia by intratracheal instillation [5,18]. However, the mechanism of apoptosis induced by SNPs needs to be further explored. According to the air quality guidelines of the World Health Organization (WHO) [19], the mice were exposed to SNPs (110 nm, spherical) with 12.5 (a low dose) and 25.0 and 50.0 mg/kg bw (high doses) by intratracheal instillation in vivo. We found that 12.5 mg/kg SNPs had no significant toxicity, while 25.0 and 50.0 mg/kg SNPs caused ovarian granulosa cell apoptosis, leading to follicular atresia (Figure S2), which is consistent with our previous study [18].

To reveal the possible mechanisms of apoptosis, lethal doses of SNPs were used and the relationship between autophagy and apoptosis induced by SNPs was revealed in primary cultured ovarian granulosa cells in vitro. According to the calculation of the real exposure levels of SNPs in vivo, the doses of 0–262.8 µg/mL were compared in vitro [20]. Consistent with previous studies [13,21], we found that SNPs were internalized into ovarian granulosa cells and mainly distributed in the lysosomes (Figure 1). In addition, 0–150 µg/mL SNPs did not have significant toxicity, while above 300 µg/mL SNPs decreased cell viability and increased apoptosis (Figure 2). In order to explore its mechanism of apoptosis, lethal doses of SNPs were used in vitro. SNPs were reported to activate apoptosis via the mitochondria pathway, such as human liver (HepG2) cells, glioblastoma (LN229) cells, and neuroblastoma (SH-SY5Y) cells [22,23,24]. Our results showed that SNPs induced apoptosis via the mitochondria pathway in ovarian granulosa cells, which regulated the BAX/BCL-2 ratio and caspase-3 level (Figure 4). BCL-2 and BAX cooperate in apoptosis via the regulation of mitochondrial membrane permeabilization. BAX destroys the integrity of the mitochondrial outer membrane, leading to cytochrome c release and apoptosis initiation [25,26,27]. BCL-2 controls BAX activation and prevents the release of cytochrome c directly or indirectly from the mitochondria [28,29]. Hence, our results indicate that SNPs induce apoptosis via the mitochondria-mediated caspase-dependent apoptotic cascade in ovarian granulosa cells.

In addition to apoptosis, SNPs also induce autophagy. Here, our results show that Stöber SNP exposure activated autophagy at both cytotoxic and noncytotoxic levels. We demonstrated that SNPs enhanced the level of BECLIN-1 and promoted LC3-I conversion into LC3-II (Figure 3). BECLIN-1 interacts with the class III-type phosphoinositide 3-kinase (PI3K) to form autophagosomes [30]. LC3, as a diagnostic marker of autophagy, cooperates in cargo delivery and autophagosome formation and development [31]. The high doses of SNPs increased the P62 level (Figure 3), indicating that SNPs inhibit autophagosome degradation and block autophagic flux at high doses in ovarian granulosa cells. P62 is involved in cargo delivery to the autophagosome by binding ubiquitinated proteins and LC3-II as well as degradation by autophagy [32]. So far, numerous studies have mainly focused on the roles of SNPs in autophagy activation induced by SNPs rather than in degradation. SNPs are reported to activate autophagy, leading to cell death in the HepG2 cells [6] and thuscausing endothelial dysfunction and apoptosis in HUVECs [33,34], thereby alleviating inflammation and inhibiting apoptosis in RAW 264.7 cells [35,36]. In addition to autophagy activation, the effects of SNPs on autophagic dysfunction have been more systematically investigated. SNPs inhibit autophagic flux and cause lysosomal dysfunction in HeLa cells [15]. SNPs inhibit autophagosome degradation to lead to autophagy dysfunction via lysosomal impairment in hepatocytes [13]. Recently, SNPs were demonstrated to induce lysosome impairment and autophagy dysfunction [14]. Consistent with the previous studies, we found that SNPs blocked autophagic flux, resulting in autophagy dysfunction. To further demonstrate whether SNPs blocked autophagic flux, SNPs combined with BAFA1, which prevented the fusion between autophagosomes and lysosomes as well as lysosomal acidification [37,38], significantly enhanced the P62 level and the transfer of LC3-I to LC3-II (Figure 5). Collectively, these results suggest that SNPs activate autophagy while perturbing autophagic flux at high-dose levels.

Autophagy is a lysosome-dependent cellular degradation process [39]. Once lysosomes are impaired, autophagic flux is blocked. To test whether SNP-induced autophagy dysfunction is related to lysosomal impairment, we evaluated the acidity of lysosomes. In this study, as expected from the results of CQ treatment [40], SNP exposure did not decrease the acidity of lysosomes as it rapidly increases LysoTracker Red puncta staining (Figure 7). Furthermore, we examined the localization of CTSD, a lysosomal aspartic protease, which is mainly expressed in the lysosome [41]. Due to lysosomal rupture, CTSD is released into the cytosol and induces cell death [42]. We found that CTSD was translocated from the lysosome to the cytosol, which was diffuse and cytoplasmic, and the level of the mature form of CTSD was decreased after SNP exposure (Figure 7), indicating that SNPs inhibited the lysosomal function.

Autophagy dysfunction has been recognized to contribute to apoptosis [13,14]. To determine the relationship between autophagy and apoptosis induced by SNPs, we assessed the cell apoptotic rate by flow cytometry pretreatment with RAP, 3-MA, and CQ, respectively. Our results showed that apoptosis induced by SNPs was significantly increased by RAP and CQ treatment, while it was decreased by 3-MA treatment, indicating that autophagy activation and dysfunction induced by SNPs enhanced apoptosis in ovarian granulosa cells (Figure 5). To further demonstrate whether autophagy induced by SNPs enhanced the mitochondria-mediated caspase-dependent apoptosis, we measured the level of the apoptosis-related proteins. The results showed that SNPs, combined with RAP or CQ, significantly increased the BAX/BCL-2 ratio, while 3-MA significantly decreased them (Figure 5), indicating that both autophagy induction and the autophagic flux blockage had a pro-apoptotic role in cytotoxicity induced by SNPs in ovarian granulosa cells.

The inhibition of autophagy formation significantly decreases apoptosis induced by SNPs. As we know, BECLIN-1 cooperates in the formation of autophagy. BECLIN-1 induces autophagosome formation via the formation of a complex with PI3K [43]. We found that the knockdown of BECLIN-1 decreased the LC3-II level (Figure 6), indicating that SNP-induced autophagy was inhibited. BECLIN-1 is discovered due to the interaction with BCL-2 [44]. The pro-autophagic activity of BECLIN-1 is inhibited by BCL-2 [45]. BECLIN-1 was also reported to inhibit cell death induced by starvation in the human SH-SY5Y cells [46]. Collectively, our results indicate that BECLIN-1 depletion inhibits autophagy and apoptosis induced by SNPs in ovarian granulosa cells.

**Figure 9:**
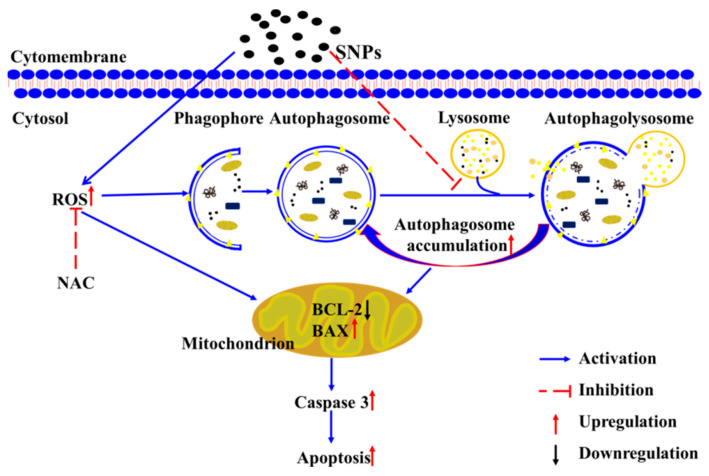
The putative schematic representation of the molecular mechanisms involved in autophagy and apoptosis induced by SNPs.

Excessive ROS production is widely accepted as one of the main toxic mechanisms caused by SNPs [6,7]. The excessive generation of ROS is reported to induce autophagy and eventually lead to cell apoptosis via the induction of oxidative stress [10]. Oxidative stress contributes to the deterioration in oocyte quality and a decline in fertility [47]. Liu et al. reported that oxidative stress cooperates in cell apoptosis in rat ovarian granulosa cells [48]. In addition, Siddique et al. found that oxidative stress causes cell apoptosis in ovarian granulosa and theca cells [49]. Recently, Jalouli et al. demonstrated that excessive ROS causes defective autophagy-related apoptosis in developing rat ovaries [50]. To determine the correlation between ROS generation and autophagy and apoptosis induced by SNPs, NAC, an antioxidant, was used to determine the potential role of ROS. Our results showed that NAC significantly decreased autophagy and apoptosis induced by SNPs via the detection of the BAX/BCL-2 ratio, cleaved caspase-3, and LC3-II levels (Figure 8). Collectively, these results suggest that SNPs activate autophagy and induce apoptosis via the production of excessive ROS. A diagram of the mechanisms involved in cytotoxicity induced by SNPs from the evidence we obtained is depicted in Figure 9.

## 4. Materials and Methods

### 4.1. Reagents and Animals

The reagents 3-MA (M9281), CQ (C6628), and Rap (V900930) were obtained from Sigma-Aldrich (St. Louis, MO, USA). LysoTracker red DND-99 dyes (C1046) and TUNEL kit (C1088) were obtained from Beyotime Biotechnology (Shanghai, China). NAC (HY-B0215) was obtained from MedChemExpress (Shanghai, China).

Female 21- and 28-day-old ICR mice were obtained from the Comparative Medicine Center of Yangzhou University (Yangzhou, Jiangsu, China). The mice were maintained under the conditions of 23–25 °C and 12-h light/12-h dark cycle. The treatment procedures were approved by the Ethics Committee of Yangzhou University (License number: 202103322).

### 4.2. Characterization of SNPs

The Stöber method was used for synthesizing SNPs as described previously [11]. The morphology of SNPs was assessed by transmission electron microscopy (TEM, Royal Philips, Amsterdam, The Netherlands). Approximately 700 nanoparticles were used to analyze the average diameter of SNPs from random images of TEM via Image J software (National Institutes of Health, Bethesda, MD, USA). The zeta potential and hydrodynamic sizes in 10 mM NaCl were tested with a Zetasizer (Malvern Instruments Ltd., Worcestershire, UK).

### 4.3. Animal Treatment with SNPs In Vivo

Female 28-day-old ICR mice were treated with SNPs according to our previous study [16]. Briefly, all the mice had free access to about 5 g of food and unlimited purified water. After 7 days of acclimation, 32 female ICR mice were weighed and randomized into four groups (number = 8 mice per group). Control mice were treated with 0.2 mL of sterile normal saline (0.9% *w/v* NaCl, control group) by intratracheal instillation. SNPs were injected into the mice with 12.5, 25.0, and 50.0 mg/kg b.w. concentrations (SNP groups) diluted in saline (0.2 mL) for two days (once per day) under the same condition. After treatment for 15 days, animals were euthanized using 5% isoflurane and one ovary from each mouse was isolated, preserved in 4% paraformaldehyde (PFA), and used for further histological examination.

### 4.4. Hematoxylin Eosin (H andE) Staining

Isolated ovaries were fixed in 4% PFA, dehydrated by washing them with increasing ethanol concentration gradient solutions, embedded in molten paraffin, sectioned on Leica RM 655 Rotary Microtome, placed onto glass slides, and stained with hematoxylin and eosin for histological examination with a digital microscope (BA400, Motic, Amoy, China).

### 4.5. TUNEL Staining

The detection of apoptosis in the ovaries was carried out using a TUNEL kit. The sections were dewaxed in xylene, rehydrated, and incubated with proteinase K solution (20 µg/mL) and terminal deoxynucleotidyl transferase (TdT) mix with fluorescein isothiocyanate (FITC) reaction solution. Next, after counterstaining with DAPI, the sections were mounted by anti-fade mounting medium and imaged using a TCS SP8 STED laser scanning confocal microscope (LSCM, Leica, Wetzlar, Hessen, Germany).

### 4.6. Primary Ovarian Granulosa Cell Culture and SNP Exposure In Vitro

Female 21-day-old ICR mice (10 female ICR mice per experiment) were used to culture ovarian granulosa cells in vitro. To stimulate follicular maturity, each mouse was injected with 5 IU of pregnant mare serum gonadotropin (PMSG, Sansheng, Ningbo, Zhejiang, China) for 48 h via intraperitoneal injection. Next, the mice were euthanized using 5% Isoflurane, the ovaries were quickly excised, and the mature follicles were punctured for the collection of ovarian granulosa cells. After filtration and centrifugation, the collected cells were suspended in fresh DMEM/F12 medium supplemented with 10% fetal bovine serum (FBS, Invitrogen, Carlsbad, CA, USA), 100 units/mL penicillin, 100 g/mL streptomycin, and distributed into 60 mm culture plates at a density of 1 × 10^6^ cells/dish and cultured at 37 °C in a 5% CO_2_ atmosphere. The purity of ovarian granulosa cells was detected by the staining of FSHR, as described previously [18]. To investigate the cytotoxicity of SNPs, cell viability and apoptosis were measured after SNP exposure.

### 4.7. SNP Cellular Internalization in Ovarian Granulosa Cells

Ovarian granulosa cells were exposed to 300 μg/mL of SNPs for 24 h. After washing with PBS to remove the excess SNPs, the cells were trypsinized, harvested, and fixed in glutaraldehyde. Next, after three rinses with PBS and dehydration through a graded ethanol series, the cells were embedded in epoxy resin. Ultrathin sections (50 nm) were stained with lead citrate and uranyl acetate and imaged using HT7800 TEM (Hitachi, Japan).

### 4.8. Measurement of Cell Viability

Cell viability was measured by CCK-8 assay (New Cell and Molecular Biotech, Suzhou, Jiangsu, China). According to a previousreport [20], the real exposure levels of SNPs are at 0–262.8 μg/mL in vitro doses, so the dose of SNPs at 150 μg/mL used in this study is close to the possible oral exposure in real life. Furthermore, we demonstrated that the SNPs induce granulosa cell apoptosis at the dose of 300 μg/mL [18]. To reveal the possible mechanisms of apoptosis, lethal doses of SNPs (above 300 μg/mL) were used in this study. The cells were seeded into 96-well plates with 3 × 10^4^ cells/well and then cultured with SNPs at doses of 150, 300, 450, 600, 750, 900, and 1050 µg/mL. After exposure for 24 h, the cells were incubated with 10 µL of CCK-8 in each well for 2 h at 37 °C. The absorbance was measured on a Model 680 microplate reader at the wavelength of 450 nm (Bio-Rad, Hercules, CA, USA).

### 4.9. Cell Apoptosis Assay

The cultured ovarian granulosa cells were exposed to SNPs at doses of 150, 300, and 600 µg/mL. After exposure for 24 h, the apoptotic rate was determined using an annexin V-FITC/PI apoptosis detection kit (KeyGen Biotech, Nanjing, Jiangsu, China). Briefly, granulosa cells were harvested, centrifugated, and resuspended in a binding buffer containing propidium iodide (PI) and annexin V-FITC for 15 min. Flow cytometry was performed using an EPICS Altra flow cytometer (Beckman Coulter Cytomics Altra, Brea, CA, USA).

### 4.10. Cell Transduction with Beclin-1 Lentiviral shRNA Vector (shBeclin1)

Beclin-1 lentiviral shRNA sequences were designed and are listed in Table 1. shRNA vectors for Beclin-1 (shBec1-1 and shBec1-2) and a control (shNC) were constructed. The shRNA lentiviruses were packaged according to the previous methods [51]. Briefly, the shRNA vectors and packaging vectors (pGag/Pol, pRev, and pVSV-G) were transfected into HEK 293T cells for 16 h and then the medium was replaced by conditioned medium, which consisted of Advanced DMEM medium, 2% FBS, 0.01 mM cholesterol, 0.01 mM egg lecithin, and 1 × chemically defined lipid concentrate used for the production of lentiviruses. After culturing for 48 h, the conditioned medium was harvested, purified, and filtered. Ovarian granulosa cells were transduced with shBec1-1, shBec1-2, and shNC lentiviral particles (multiplicity of infection (MOI) = 20) for 48 h and then exposed to SNPs for further experiments.

### 4.11. LysoTracker Red Staining

Ovarian granulosa cells were seeded on cover slips in 24-well plates and then exposed to both 300 µg/mL SNPs and 100 μM CQ. Next, the cells were incubated with 75 nM LysoTracker Red and cultured at 37 °C for 30 min in the dark. After washing with PBS three times, the cells were imaged using an LSCM (Wetzlar, Hessen, Germany). The number and size of lysosomes in each cell were analyzed using Image J software.

### 4.12. Immunofluorescence Staining

Ovarian granulosa cells were seeded on sterile cover slips in 24 well plates and then exposed to both 300 µg/mL SNPs and 100 μM CQ. Next, the cells were fixed in 4% PFA, permeabilized with PBS containing 0.5% Triton X-100, blocked with 5% bovine serum albumin (BSA), and incubated with rabbit anti-CDST antibody (ab75852; 1:1000 dilution, Abcam, Cambridge, MA, USA) overnight at 4 °C. After washing with PBS, the cells were incubated with secondary fluorescent-conjugated goat anti-rabbit antibody (ab150077, 1:1000 dilutions, Abcam) for 1 h followed by incubation with DAPI for nuclear staining for 10 min in the dark at room temperature. Finally, the cellular fluorescence was observed and imaged using LSCM.

### 4.13. Western Blot

Ovarian granulosa cells were harvested, collected into cold EP tubers, resuspended in lysis buffer, centrifuged, and the supernatants were collected. The concentration of protein was determined by a BCA protein assay kit (KeyGen Biotech, Nanjing, Jiangsu, China). After protein denaturation, a total of 20 μg of protein was electrophoresed in SDS-PAGE. Next, the proteins were electrotransferred to polyvinylidene difluoride (PVDF) membranes. After transfer and blockage with 10% nonfat dry milk solution for 1 h, the PVDF membranes were incubated overnight with primary antibodies for cleaved caspase-3 (9664; 1:1000 dilution, Cell Signaling Technology (CST), Danvers, MA, USA), BCL-2 (ab182858; 1:1000 dilution, Abcam), BAX (ab32503; 1:1000 dilution, Abcam), CTSD (ab75852; 1:1000 dilution, Abcam), SQSTM1/P62 (A19700; 1:500 dilution, ABclonal, Wuhan, Hubei, China), BECLIN-1 (A11761; 1:500 dilution, ABclonal), LC3 (L7543; 1:1000 dilution, Sigma-Aldrich), and β-actin (AC026; 1:2000 dilution, ABclonal) at 4 °C, respectively. After washing with TBST, the membranes were no longer present as demonstrated upon incubation with secondary HRP-conjugated goat anti-rabbit (7074; 1:5000 dilution, CST) and anti-mouse (7076; 1:5000 dilution, CST) antibodies, respectively. Protein blots were scanned using a ChemiDoc XRS + System (Bio-Rad, Hercules, CA, USA).

### 4.14. Statistical Analysis

Data were expressed as mean ± standard error of the means (SEM) from independent experiments, which were performed in triplicate and repeated three times. Statistical significances of the differences between control and treated groups were analyzed by ANOVA and followed by LSD. Significance was accepted at *p* < 0.05.

## 5. Conclusions

In summary, our study demonstrates that SNPs activate autophagy and cause autophagic dysfunction. Meanwhile, SNPs induce cell apoptosis via the activation of the mitochondria-mediated caspase-dependent apoptotic pathway. Furthermore, autophagic dysfunction induced by SNPs enhances cell apoptosis in ovarian granulosa cells. Taken together, SNPs induce cell apoptosis via autophagic dysfunction in ovarian granulosa cells, leading to follicular atresia.

## Figures and Tables

**Table 1:** Sequences of shRNAs.

Interference Fragment	Synthetic Primers	Sequence (5′–3′)
shBec-1	shBec-1F	GATCCCAGTCTCTGACAGACAAATCTCTCGAGAGATTTGTCTGTCAGAGACTGTTTTTG
	shBec-1R	AATTCAAAAACAGTCTCTGACAGACAAATCTCTCGAGAGATTTGTCTGTCAGAGACTGG
shBec-2	shBec-2F	GATCCCAATAAGATGGGTCTGAAGTTCTCGAGAACTTCAGACCCATCTTATTGTTTTTG
	shBec-2R	AATTCAAAAACAATAAGATGGGTCTGAAGTTCTCGAGAACTTCAGACCCATCTTATTGG
shNC	shNC-F	GATCCGATGAAATGGGTAAGTACACTCGAGTGTACTTACCCATTTCATCTTTTTG
	shNC-R	AATTCAAAAAGATGAAATGGGTAAGTACACTCGAGTGTACTTACCCATTTCATCG

## Data Availability

Data available on request due to restrictions eg privacy or ethical.

## Supplementary Materials

The following supporting information can be downloaded at: https://www.mdpi.com/article/10.3390/ijms24065189/s1.
