# Supplementary material for: Silica Nanoparticles Promote Apoptosis in Ovarian Granulosa Cells via Autophagy Dysfunction
Source: Int J Mol Sci. 2023 Mar 8;24(6):5189. doi: 10.3390/ijms24065189 (PMC10049489; doi:10.3390/ijms24065189)
Supplement: Supplementary file 1 [file ijms-24-05189-s001.zip › ijms-2216293-supplementary.pdf]

Supplementary material

Silica nanoparticles promote apoptosis of ovarian granulosa cells via autophagy dysfunction

Zhen Zheng[1,2,3†],Wenlong Zuo[1†], Rongrong Ye[1,2,3], Jason William Grunberger[4,5], Nitish Khurana[4,5], Xianyu Xu[1,2,3], Hamidreza Ghandehari[4,5,6], Fenglei Chen[1,2,3*]

[1] Institute of Comparative Medicine, College of Veterinary Medicine, Yangzhou University, Yangzhou 225009, China

[2] Jiangsu Co-innovation Center for Prevention and Control of Important Animal Infectious Diseases and Zoonoses, Yangzhou University, Yangzhou 225009, China

[3] Joint International Research Laboratory of Agriculture and Agri-Product Safety, the Ministry of Education of China, Yangzhou University, Yangzhou 225009, China

[4] Department of Molecular Pharmaceutics, University of Utah, Salt Lake City, Utah, United States

[5] Utah Center for Nanomedicine, University of Utah, Salt Lake City, Utah, United States

[6] Department of Biomedical Engineering, University of Utah, Salt Lake City, Utah, United States

*Corresponding author: E-mail: flchen@yzu.edu.cn; Tel: +86-514-87979030; Fax: +86-514-87972218.

[†] These authors contributed equally to this work.

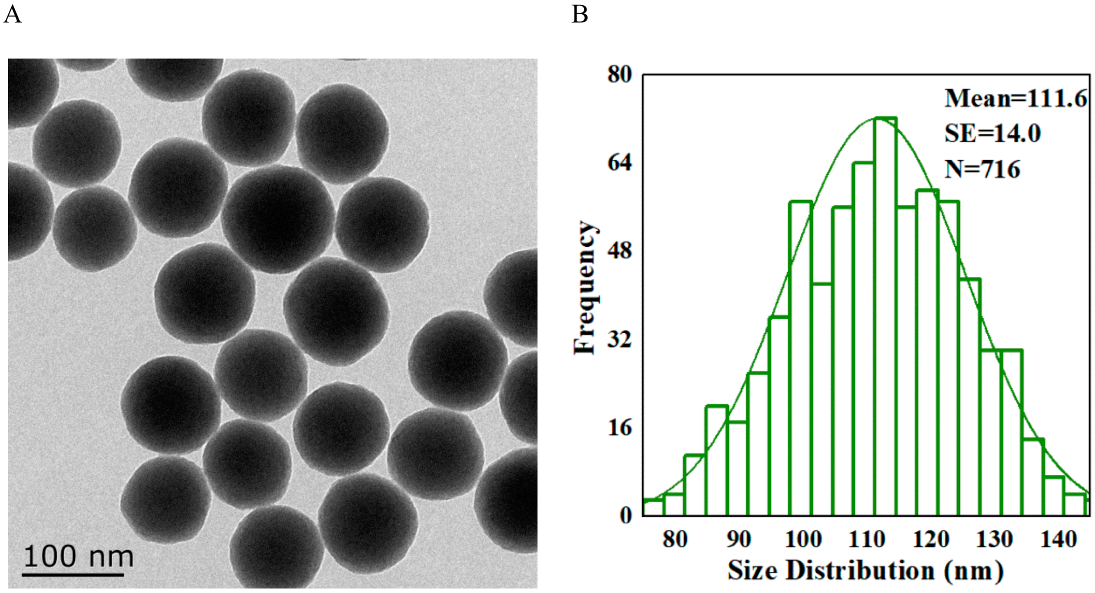

Figure S1. Morphology of SNPs. (A) Representative TEM image of SNPs synthesized by the Stöber method. (B) Size distribution histograms of SNPs analyzed by Image J software in A.

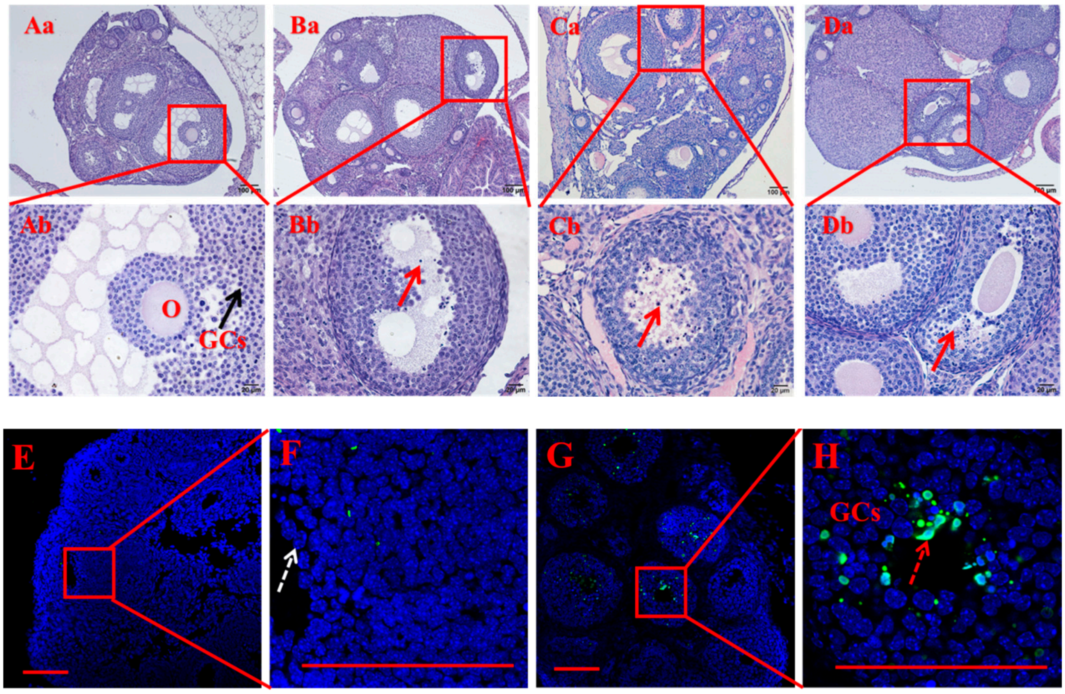

Figure S2. Effects of SNPs on the histological structure of the ovary. (Aa) Representative H&E image of the ovary in the control group. (Ab) Selected area in Aa. (Ba) Representative H&E image in the 12.5 mg/kg SNP group. (Bb) Selected area in Ba. (Ca) Representative H&E image in the 25.0 mg/kg SNP group. (Cb) Selected area in Ca. (Da) Representative H&E image in the 50.0 mg/kg SNP group. (Db) Selected area in Da. Black arrow points to the healthy granulosa cells.

Red arrow points to the apoptotic granulosa cells. (E) Representative LSCM image in the control group. (F) Selected area in E. (G) Representative LSCM image of the ovary in the 50.0 mg/kg SNP group. (G) Selected area in H. Green fluorescence points to TUNEL staining, and blue fluorescence points to DAPI staining. Red dotted arrow points to the apoptotic granulosa cells. White dotted arrow points to the healthy granulosa cells. GCs, granulosa cells; O, oocyte. Scale bars, 100 μm (Aa, Ba, Ca, Da, and E-H) and 20 μm (Ab, Bb, Cb, and Db).
